# Surgery

**Published:** 1878-03

**Authors:** 


### Summary.

### SURGERY.

GURJUN BALSAM IN GONORRHŒA.—*Journal de Médecine, December*, 1877.—M. Vidal is the first in France who has studied the applications of this new remedy, whose remarkable properties will certainly bring it into use speedily. It is obtained from several resinous trees in the Indian Archipelago, is very abundant, and the price is moderate.

Gurjun balsam has been successfully employed for leprosy by several English physicians in India, and Vidal has also had good results from its use in the Hôpital Saint Louis. But it is especially in gonorrhœa that it renders the greatest service. M. Deval, a student of Vidal, gives ten cases as proof of its value, and his testimony is corroborated by Maurice and others. The duration of treatment varied from ten to twenty days, the duration being shorter in proportion as the patient had passed the inflammatory stage. Vidal's formula is :

| | | |
|---|---|---|
| Gurjun balsam (wood oil), | | |
| Acacia, | ā ā | 4 grammes. |
| Infusion of anise seed, | 40 | " |

To be taken before meals. It was not necessary to increase the dose, which is perfectly well tolerated, the only effect being to cause one or two stools, two hours after the meal. When the dose was increased, no more than eight grammes were given. Sometimes at first a little nausea was produced, but this speedily disappeared. Vidal gives a little wine after the potion, which makes it better tolerated. No change in diet is necessary. Besides the potion, a liniment of equal parts of the balsam and limewater, applied by means of tampons, was used in women with vaginitis; the tampons were left in the vagina twenty-four hours. A cure was always rapid in women. Its advantages over copaiba are its more rapid and certain action; it does not produce erythema, and it does not give to the breath the tell-tale odor of copaiba. Its local action in vaginitis and balanitis is also excellent.

Lister's Antiseptic Dressing. Surgery. Practical Results. Prof. Létiévant. (*Lyon Médicale*, Dec. 16, 1877.)

The author introduced this method into his service in 1869, but being dissatisfied with its results abandoned it. He reintroduced it with new modifications, two years ago, and has had the most excellent results. He has sought to follow as exactly as possible Lister's great fundamental principle not to allow the external air to come in contact with the wound, but to keep it constantly surrounded with an atmosphere of carbolic acid vapors. He has used the various atomizers for this purpose, at the moment of operating and dressing. He does not use the substances for dressing that Lister does, but employs fine, waxed taffeta as a protecting envelope, then cotton passed through potash or soda lye, dried and finally charged at the moment of dressing with a two and one-half per cent solution of carbolic acid. These are kept in place by a bandage, also saturated with the same solution. A layer of dry cotton envelopes the whole.

Three great facts have been proved by his two years experience: 1st, purulent infection has not reappeared in his service; 2nd, grave complicated wounds have healed with much greater facility; 3rd, immediate union attempted after operations has nearly always been followed by success. Besides, there has been greater cleanliness of the wounds, less suppuration, and disappearance of infectious odors of the wounds, wards, etc.

Before the introduction of this method purulent infection was frequent at the Hôtel-Dieu, sometimes epidemic. Since its introduction two years ago, not a single case has occurred in the service of M. Létiévant. There have been more than 1,500 operations during these two years, the range of cases has been the same as previously and nothing has changed except the plan of dressing. The author gives a list of 20 serious compound fractures in which there were eighteen cures and two deaths.

Such satisfying results were not known before. Amputation was the rule in compound fractures of the leg, especially if the joint was opened. Now it is the exception. The antiseptic dressing not only allows the conservation of the limb in grave compound fractures, but it authorizes a trial of new operations heretofore regarded impracticable. As an example the author mentions a case of chronic suppurative osteo-arthritis of the wrist, in which by means of two lateral incisions he extirpated all the carpal bones, resected the radial and cubital styloid apophyses, and the superior extremity of the second metacarpal bone. Moderate suppuration followed and there was no complication. Several other grave cases are mentioned, all showing the benefits of the antiseptic treatment.

The good effect of the new dressing has not only been shown in large wounds, accidental or surgical, but its value has appeared still more striking in efforts toward immediate union in serious wounds. Formerly repeated efforts were rarely followed by success. Now, nearly all are crowned with success. Among them may be mentioned operations for cancer of breast, neck, epitheliomata of lip, vesico-vaginal fistula with included neck of uterus in bladder, amputations of thumb, great toe, finger, arm, leg, thigh, strangulated hernias, tumors, neurotomies, etc. The author sums up the conditions necessary to obtain immediate union after operations, they are: 1st, Operate under a cloud of carbolic vapors; 2d, secure exsanguification of the member if possible, or at least a perfect digital compression; 3d, operate rapidly, that the air may be in contact with the wound as short a time as possible; 4th, twist the arteries, or tie with cat-gut, which may absorb; 5th, bring the flaps together closely, so that no oozing may take place from their surfaces during the suturing; 6th, use the interrupted metallic suture; 7th, dress according to his modification of Lister's method; 8th, sustain the dressing by a thick layer of cotton, destined to keep the stump warm and under a gentle pressure. The author would leave drainage tubes at the angle of wounds only a few days after the operation.

The author accords the greatest importance to Lister's method as practiced by him. Without, he has scarcely ever seen immediate union; since its use it has been so common that he does not hesitate to regard it as the rule after operations.

To sum up: Immediate union in cases where one would not have dared to hope for it; suppression of purulent infection; conservation of parts in grave cases where amputation was formerly always performed.

Tracheotomy in Diphtheria.—By W. H. Quill. (*Medical Press and Circular*, Jan., 1878.) Dr. Q. read before the Surgical Society of Ireland a paper on this subject. The patient was a healthy boy five and a half years old. The fourth day tracheotomy was decided on, but before the operation could be accomplished a frightful paroxism of dyspnœa came on and the child was apparently dead. The trachea was immediately opened, and a quantity of bloody mucus and unmistakable pieces of diphtheritic false membrane gushed out. The change was marvelous, the natural color returned, and, with a long-drawn inspiration, the child opened its eyes.

Obré's double tracheotomy tube was inserted, and held securely in place by tapes passed around the neck. Through this the patient breathed freely, and being placed in bed, fell into a calm sleep.

No difficulty was experienced in the operation. Care was taken not to wound any of the veins in the line of incision, so the hemorrhage was slight.

The case progressed favorably until the second day after the operation, when dyspnœa suddenly supervened. Dr. Johnson, who happened to be present, succeeded, by means of a long feather, in freeing the obstructed tube. A violent expiration followed, during which a perfect cast in false membrane of the lower portion of the trachea and upper portion of the right bronchus was expelled, giving immediate relief. This cast measured four inches in length. On the 9th day difficulty of swallowing gradually came on, and for several days the child was nourished entirely by nutrient enemata.

This case, Dr. Q. thinks, will help to dispel the idea "that tracheotomy must be looked upon as simply palliative when false membranes have extended into the trachea." For the formation of false membrane seems *to be arrested by the operation*, and the introduction of a large tube close to the seat of the disease enables the false membrane to be discharged with greater facility than if they had to traverse the entire length of the trachea and larynx. Moreover, the artificial opening gives physiological rest to the larynx—a point of no mean value when one remembers its structure, and the ill effects that must result from its abnormal constant activity, and the frequent forcible contact of pellets of diphtheritic deposit.

He advocates the operation in all cases where well directed medical treatment is evidently failing, evidenced principally by more or less lividity of the face, by strong and deep depressions of the epigastrium at each inspiration, and by working of the nostrils.

He also considers it incumbent on the surgeon to perform the operation, even though the chance of ultimate recovery be infinitesimal. He here alludes to those so-called cases of bronchitic diphtheria, for more than one case of recovery has been recorded even after the false membrane molded to the tubes has been ejected, and if the only result of the operation was euthanasia, it would still be his duty to lessen the agonizing death throes.

While making full allowance for skill and coolness during the operation, much of the ultimate success, it must be acknowledged, depends on the after management of the case, for it is an interesting fact that a large proportion of deaths following tracheotomy are due, not to diphtheria, but to bronchitis or pneumonia, which follows the operation, as these affections are in great part attributable to the admission of air to the lungs, which has not received its normal degree of warmth in its circuit through the nostrils, mouth and larynx. He holds that the necessity for the air receiving the essential degree of warmth is of equal importance with keeping the tube clear.

